# Experiences of Peri-partum Urinary Incontinence from a Women’s and Health Care Perspective: A Qualitative Study

**DOI:** 10.1007/s10995-023-03631-6

**Published:** 2023-03-29

**Authors:** Heidi F. A. Moossdorff-Steinhauser, Inge Houkes, Bary C. M. Berghmans, Marc E. A. Spaanderman, Esther M. J. Bols

**Affiliations:** 1 School for Public Health and Primary Care (CAPHRI), Maastricht University, P.O. Box 616, 6200 MD Maastricht, The Netherlands; 2 Department of Social Medicine, Maastricht University, Maastricht, The Netherlands; 3 Pelvic Care Center Maastricht, CAPHRI, Maastricht University Medical Centre (MUMC+), Maastricht, The Netherlands; 4 Department of Obstetrics and Gynaecology, MUMC+, Maastricht, The Netherlands

**Keywords:** Beliefs, Health care professional, Post-partum, Pregnancy, Urinary incontinence

## Abstract

**Objectives:**

Urinary incontinence (UI) is highly prevalent peri-partum. To gain more understanding regarding the gap between the prevalence of UI and actual help seeking behaviour of peri-partum women, this study aims to understand, (1) how peri-partum women experience UI and which factors influence these experiences and (2) the perspective of health care professionals on UI during pregnancy, and the first year after childbirth.

**Methods:**

A qualitative approach was used, using semi-structured interviews with adult pregnant and up to 1 year post-partum women and a focus group with health care professionals (HCP’s) involved in the care of pregnant and post-partum women. Thematic analysis was used to analyse the data.

**Results:**

Six pregnant and seven post-partum women were included. Nearly all of these women expressed to be not, or only slightly bothered by their UI and accept it as a result of pregnancy and/or delivery. They were surprised because they were unaware that UI could be a problem. None of the HCP’s routinely asked about the presence of UI during pregnancy. At the post-natal check at 6 weeks post-partum, UI is still not a standard question for the majority of the gynecologists and registrars in contrast to the midwives.

**Conclusions for Practice:**

The interviewed women with UI during pregnancy and the first year after childbirth were surprised but hardly bothered by their UI and accept it as part of being pregnant or as a result of the delivery. HCP’s do not routinely discuss UI during pregnancy or post-partum.

## Significance

*What is already known on this subject?* Peri-partum women with urinary incontinence often believe it is normal and will resolve by itself over time. Health care professionals in peri-partum care, although knowledgeable on urinary incontinence, do not standardly discuss urinary incontinence in The Netherlands. A majority of peri-partum women do not seek professional help.

*What this study adds?* The beliefs peri-partum women have on urinary incontinence probably originates from information from a health care professional, friend or the internet. To disclose their urinary incontinence, women need to be timely and structurally asked by their health care professional. A lack of time is one of the reasons why health care professionals do not standardly discuss this topic.

## Objectives

Pregnancy and delivery are well known risk factors for the development of urinary incontinence (UI) during pregnancy and post-partum (Rortveit et al., 2001). The prevalence of UI, defined as the involuntary loss of urine rises from 55% in the first to 70% in the third trimester (Moossdorff-Steinhauser et al., 2021a). In the first year post-partum, 31% of women report UI with no significant difference in prevalence numbers between 6 weeks, 6 months and 12 months post-partum (Borello-France et al., 2006; Hansen et al., 2012). Although the prevalence numbers of UI during pregnancy and up to 1 year post-partum are high, the reported corresponding experienced bother of UI appears to be low to moderate (Hansen et al., 2012; Martin-Martin et al., 2014; Martínez Franco et al., 2014; Moossdorff-Steinhauser et al., 2021a). There are indications that the level of perceived bother influences help-seeking behaviour for UI (Hägglund et al., 2001). Two recently published surveys on the prevalence, experienced bother, beliefs, and help-seeking behaviour in the peri-partum period in The Netherlands, confirmed the high prevalence numbers in combination with low to moderate experienced bother (Moossdorff-Steinhauser et al., 2021b, c). However, these studies also showed that only 13% of pregnant women and 26% of women with UI between 6 weeks and 1 year post-partum sought help for their UI. A remarkable result is that nearly 40% of non-help seeking pregnant women and 49% of non-help seeking post-partum women believe that their UI will resolve spontaneously or improve greatly after delivery or over time (Moossdorff-Steinhauser et al., 2021b, c). Even though health care professionals who provide peri-partum care are knowledgeable of risk factors of pelvic floor dysfunctions like UI, it is not common practice to educate and counsel peri-partum women on UI (Cooke et al., 2018). These results demonstrate a persistent knowledge gap among women regarding natural recovery or improvement of UI and counselling by health care professionals (Buurman & Lagro-Janssen, 2013). Pregnant women are not aware of the fact that UI in pregnancy results in a two to six fold risk of UI post-partum (Burgio et al., 2003) whereas up to 91% of post-partum women with UI still report UI after 12 years (Diez-Itza et al., 2020). Existing guidelines recommend pelvic floor muscle exercises as a first line treatment option for women with UI (National Institute for Health and Care Excellence (NICE), 2019a). However, only a few women actually seek help. To gain more understanding regarding the gap between the prevalence of UI and actual help-seeking behaviour of peri-partum women it is important to understand the health beliefs of these women and their caretakers regarding UI, how peri-partum women experience their UI, and to acquire knowledge on subsequent health care management.

Therefore, this research aims to understand, (1) how peri-partum women experience UI and which factors influence these experiences and (2) the perspective of health care professionals on UI during pregnancy, and the first year after childbirth.

## Methods

### Design, Participants and Procedure

A qualitative approach, using semi-structured interviews with women and a focus group with health care professionals (HCP) was used. Adult, pregnant and up to 1 year post-partum women who gave birth to a living baby, regardless of parity, were eligible to participate. Due to the sensitive nature of the topics, women were interviewed individually in face-to-face interviews. To promote discussion on experiences and beliefs on UI (and related bother) and how they incorporate this in the approach of their patients, HCP’s involved in the care of pregnant and post-partum women like midwifes, residents and gynecologists participated in a focus group discussion.

Pregnant and post-partum women were recruited through purposive sampling. We aimed for a sample with sufficient variety regarding trimester in pregnancy and post-partum period, in order to increase transferability of the study results. Three midwifery practices in the area of Maastricht, The Netherlands, posted a social media message with general information on the study on their Facebook page. Interested women contacted the researcher by email. Subsequently, the researcher emailed the potential participant the more elaborate patient information letter and asked the woman to email a telephone number if she agreed to be contacted after a week. After a week the researcher contacted the woman to ask, if the research information was clear, if she had any questions, and if the woman was willing to participate. The HCP’s were recruited by email or personally invited to participate by MS. All HCP’s received an information letter well in time before the focus group. Each participant signed an informed consent form prior to participation. All included women received a €25 gift card for participating and the HCP’s €100 in cash for their time. An overview of participant characteristics is displayed in Table 1.

**Table 1:** Participant characteristics

Participant (pregnant women)	Age (years)	Education	Parity and gravidity (weeks pregnant)	General health	NRS UI
				(0 = worst possible, 10 = good)	
G1	33	Tertiary	P1G2 (33)	6	7
G2	24	Secondary	P0G1 (36)	7	6
G3	30	Tertiary	P0G1 (20)	8	7
G4	38	Tertiary	P1G2 (25)	9	7
G5	36	Tertiary	P1G3 (29)	8	5
G6	37	Tertiary	P2G3 (34)	7	5

Participant (post-partum women)			Parity (months post-partum)		
P1	30	Secondary	P2 (6)	9	7
P2	26	Secondary	P1 (5)	2	6
P3	29	Tertiary	P2 (10)	8	8
P4	25	Secondary	P1 (8)	7	6
P5	25	Secondary	P1 (2)	8	6
P6	38	Secondary	P1 (2)	9	8
P7	25	Secondary	P1 (9)	8	7

Participant (health care professional)	Type of health care professional	Hospital or privately practice
H1	Gynecologist	Hospital
H2	Midwife	Private practice
H3	Gynecologist	Hospital
H4	Resident gynecology	Hospital
H5	Resident gynecology	Hospital
H6	General practitioner specialized in urogynaecology	Private
H7	Midwife	Hospital
H8	Midwife	Hospital

*G* pregnant/gravida, *P* post-partum, *H* Health care professional, *NRS* numeric rating scale, *UI* urinary incontinence

The Medical Ethics Committee of the Maastricht University Medical Centre (MUMC +) was consulted. This committee declared that ethical approval was not necessary due to the non-invasive character of the study (MECC 019-1351).

### Data Collection

An interview guide was developed for the semi-structured face-to-face and focus group interviews, based on published literature (Cooke et al., 2018; Koch, 2006; Moossdorff-Steinhauser et al., 2021b, c; Wagg et al., 2017) and expert opinion (Tables 2 and 3). The interviews were digitally recorded. The recordings were transferred to a safe storage place on the server of Maastricht University and subsequently deleted from the recording device. Data were de-identified by giving each participant a unique code.

**Table 2:** interview guide pregnant and post-partum women

1	What does it mean for you to have urinary incontinence?
2	How does urinary incontinence influence your daily life, relations, work, sports?
3	How would you describe bother of urinary incontinence?
4	Did your health care professional ask about urinary incontinence?
	If yes, what was the advice given?
5	What actions did you take regarding your urinary incontinence?
6	What factor is important for you to seek help?

**Table 3:** Interview guide focus group

1	Who wants to tell us about discussing urinary incontinence during consultation?
2	What is the reason for discussing urinary incontinence?
3	Are there reasons for not discussing urinary incontinence?
4	What advise do you give a woman with urinary incontinence?
5	Do you think women are bothered by their urinary incontinence?

Women were visited in the privacy of their home or in one case at work by a female interviewer, HM. Visits at home offer more convenience with regard to childcare and we anticipated facilitating participation. The focus group took place in a meeting room of the local hospital. The focus group interview was done by HM together with EB who took notes and assisted if necessary. The duration of the face-to-face interviews was approximately 45 min and the focus group discussion lasted 2 h. Data collection commenced in September 2019 and was completed until saturation was reached in December 2019. Saturation was reached when no new topics emerged in the last interviews.

### Data Analysis

The recordings were transcribed verbatim by HM after every second interview to allow for constant comparison. If new topics arose during the face-to-face interviews alterations were made to the interview guide. NVIVO 12 pro was used for data analysis. Data were analysed thematically based on Braun and Clarke (Braun & Clarke, 2006). Every transcript was read thoroughly multiple times line by line and coded. The codes were reread and if necessary merged, divided, renamed and grouped in potential themes (HM). Next, mind-maps were made of the potential themes (one for the peri-partum women and one for the health care professionals) to help refine potential themes, find different levels of relationships and finally reveal the main themes. The potential themes and mind-maps were discussed and refined with EB until consensus was reached (Braun & Clarke, 2006). Themes were then discussed with the research team. The Consolidated criteria for reporting qualitative research (COREQ checklist) (Tong et al., 2007) were followed.

### Research Team

The face-to-face interviews and focus group were conducted by the first author HM as part of a PhD on UI peri-partum. She has over 20 years of clinical experience in specialized physical therapy (PT). HM also performed the primary data analyses. IH (PhD) is experienced in social medicine and qualitative research. All researchers are experienced in quantitative research. EB (PhD) is an epidemiologist, PT and has experience in project management and supervision. BB (PhD) is a clinical epidemiologist, experienced in project management and has over 35 years of clinical experience in specialized PT. MS (MD, PhD) is an obstetrician.

## Results

In total, six pregnant and seven post-partum women were included (Table 1). The pregnant women were aged between 24 and 38 years, of which two women were primi- and four were multi-gravida. The post-partum women were aged between 25 and 38 years. Five women were primi- and two multiparous. Eight HCP’s participated in the focus group discussion. The group consisted of three midwives, one working in a private practice and two in a hospital setting, two gynecologists, two residents in gynecology and a general practitioner specialized in urogynecology (Table 1).

First, the results from the peri-partum women will be discussed, followed by the results from the HCP’s.

### Peri-partum Women

Nearly all women expressed to be not or only slightly bothered by their UI. None of the participants had visited a specialized physical therapist (PT) to treat their UI. Most women accept their UI as a result of pregnancy and/or delivery. After analysis two main themes emerged: (1) feelings and beliefs regarding UI and (2) coping and adaptive strategies.

1. Feelings and beliefs regarding UI

Women with UI expressed different feelings regarding their UI for example surprise but also negative and neutral feelings. Women were surprised because they were unaware that UI could be a problem in pregnancy or post-partum, that it happened to them or that it did not resolve after delivery. Some women expressed negative feelings like: shame, fear of smell or visibility of their UI for others.

‘I expected my UI to resolve nearly completely after delivery in such a way that it would not bother me anymore.’(P5, post-partum).

‘I think I am a bit embarrassed. You are 25 and have UI already.’ (P5, post-partum).

Pregnant women more often than post-partum women indicated to have a neutral feeling.

‘There are worse things you can have during pregnancy, like high blood pressure.’ (G3, pre-partum).

Most women accept their UI as an inevitable part of being pregnant or a consequence of delivery and do not experience UI as a major problem. Often pregnant women expect it to be temporary. This belief seems to originate from a variety of beliefs based on gathered information.

‘I am 30, I have delivered a baby. That takes its toll from the body. The baby comes out vaginally. You are not left without damage.’ (G1, pre-partum).

‘The midwife told me that UI is unfortunately one of the unwanted but normal issues that is part of pregnancy and delivery.’ (P6, post-partum).

The feelings and beliefs the women expressed originated from different sources (see below).

(2) Coping and adaptive strategies

Two sub-themes emerged from this theme: (a) self-management and (b) help-seeking.

- Self-management

Different strategies were used to gather information on UI. Some women actively asked a HCP, friend or searched the internet. However, it was also mentioned that they stopped surfing the internet for information on UI because of the contradictory information found.

‘I have googled UI but you read so many different things that I stopped searching, to be honest’. (P2, post-partum).

One woman expressed that she gained information by listening to others and another woman said that if she was not asked directly about UI she would not discuss it herself.

‘I did not talk about it. A lot of people in my surroundings were pregnant as well. They did talk about it and I thought: ‘fortunately, I am not the only one with this issue’. More women have the same problem.’ (P5, post-partum).

Women with UI used different preventive and adaptive strategies for their UI. Strategies used were for instance: reducing the fluid intake, increasing micturition frequency, reducing spontaneity by for instance laughing less loud or coughing less deep, and contracting the pelvic floor muscles. The use of pads and wearing darker colour pants were also used as adaptive strategies.

‘I try to squeeze down below for as far as it goes, but also prevent sneezing. What has also changed is that I go to the toilet to urinate as soon as I feel a little bit of urge.’ (G3, pre-partum).

Two of the women did regular pelvic floor muscle exercises. These exercises were recommended by a general practitioner or a PT. Although most of the women indicated that they know that you can treat UI with pelvic floor muscle exercises, they did not perform them. Reasons were: questioning the effectiveness, being too busy with other and more urgent things. Moreover, issues like costs, travel distance and resistance against an internal palpation by a specialized PT were mentioned as barriers.

b. Help-seeking

A few women actively sought help for their UI by visiting a HCP like a general practitioner, their midwife or discussing it with the PT they were seeing for other issues.

‘When I stopped bleeding 3 weeks after my delivery, I noticed that I was losing urine. I immediately made an appointment with my general practitioner.’ (P2, post-partum).

Reasons women expressed to seek help in the future were: waiting until after delivery, an increase in the amount and frequency of urine loss, occurrence at unexpected moments or an increase in negative feelings regarding their UI.

‘I know I might lose urine when I cough or sneeze, I can live with that. I think that when I lose urine all the time I will be more bothered and then it is time to get help.’ (G3, pre-partum).

None of the participants visited a specialized PT for her UI. Pregnant women received differing advice from their HCP and also from (specialized) PT’s. Frequently, pregnant women were advised to not to perform pelvic floor muscle exercises until after delivery. One woman was quite upset that a specialized PT she had visited during her first pregnancy for pelvic girdle pain advised her not to perform the exercises until after delivery in contrast to the one she is visiting in her second pregnancy who did give her exercises.

‘When I contacted my specialized physical therapist in my first pregnancy because of my UI she told me that she could not help me. The specialized physical therapist I am visiting now (for pelvic girdle pain) thinks differently about that. So I am really disappointed.’ (G5, pre-partum).

### Health Care Professionals

None of the HCP’s routinely asked about UI during pregnancy. At the final check at 6 weeks post-partum, UI is not a standard question for the majority of the gynecologists and registrars in contrast to the midwives. For midwives working in the hospital and in private practice, this is a more common question in their final consultation.

The focus group interview with HCP’s involved in the care of peri-partum women revealed two themes: (1) lack of awareness, attention and solutions, and (2) different advice.

1. Lack of awareness, attention and solutions

The degree of awareness of UI during consultation varies considerably between HCP’s. Different reasons were provided for less awareness. The majority of the HCP’s indicated that the duration of a consultation is a big issue and that they need to prioritise.

‘We have 15 minutes for the post-partum check. If a woman had a difficult childbirth there are a lot of issues that need to be discussed. UI is not considered as important in comparison.’ (H4, registrar).

The HCP’s discussed solutions for this problem and thought that it would benefit women if they receive an information leaflet, see a poster or information on the televisions in the waiting room. Another suggestion was to discuss UI during a regular planned appointment for all pregnant women with a specialized nurse. A midwife working in a private practice has a specialized PT in her practice and she mentioned the benefits of this collaboration. The specialized PT educated her in the importance of asking about, for instance, UI, explaining the specialized PT intervention and when to refer for consultation. Some HCP’s are aware some women do not discuss issues like UI by themselves and that they need help in making it discussable.

‘You need to normalize UI, tell women that it is a common problem, but that you can do something about it.’ (H6, general practitioner).

The lack of a standard question on UI in the hospitals electronic patient file (EPR) as a reminder is also considered an important factor in not discussing UI, which was confirmed by a resident with experience in a hospital with a standard question on UI post-partum in the EPR.

‘UI is not a standard question in our EPR, which is a shame. If it was a standard question it will not be forgotten to ask.’ (H1, gynecologist).

‘I will be honest and say that it is not a question in my standard list for consultations in pregnancy. I also do not ask actively for it.’ (H5, resident).

One midwife working in a private practice has a standard question on UI in the EPR for the post-partum check. In general, the HCP’s working in the hospital agreed that a question on UI in the EPR would help them in not forgetting to ask the woman. HCP’s that actively ask post-partum women about UI often did this because they have been informed by a friend, colleague or because of their own experience. This specific information made that they became aware that UI is an important issue to discuss.

‘For me taking part in sport after my own delivery was an eye-opener, because women talked a lot easier about their ailments and it felt like being among fellow sufferers.’ (H5, midwife).

‘A friend of mine is a fitness instructor for peri-partum women, so she sees a lot of women post-partum. She said ‘don’t make the mistake and think that women will tell you about their UI. You really need to ask this question.’ (H5, resident).

(2) Different advice

The second theme ‘advice’ revealed that HCP’s give peri-partum women with UI diverse advice. Sometimes pregnant women are advised to wait and see till after delivery. Other strategies mentioned were to refer to the general practitioner, a urogynecologist or a specialized PT.

‘I refer all peri-partum women with UI to the specialized physical therapist.’ (H2, midwife).

‘If someone has a huge problem at 6 weeks post-partum then I know that pelvic floor muscle exercises are not going to help and I don’t want to ‘beat her around the bush’ so I will send her to the urogynecologist.’ (H1, gynecologist).

The discussion revealed some questions and beliefs regarding specialized PT like: how do we know that we send our patient to a properly qualified specialized PT, is there a quality register, and where do we find the location of a specialized PT? Some doubt was expressed regarding the effect of specialized PT.

‘Specialized physical therapy is more a kind of in between solution. It can ease the problem, but not solve it.’ (H1, gynecologist).

‘I have some doubts about specialized physical therapy during pregnancy for UI. On the one hand the baby has to grow and the tissue has to gain in elasticity and if I think what the specialized physical therapist wants. It is only a physiological idea.’ (H5, resident).

In conclusion, these issues suggest that HCP’s are not well aware of the current guidelines on treatment of UI.

## Discussion

This study aimed to understand on the one hand women’s experience and beliefs regarding their peri-partum UI and which factors influence these experiences, and on the other hand, learn about the perspective of HCP’s on UI peri-partum. Thematic analysis was used to analyze the interviews with the women and the focus group with the HCP’s.

Our results show that although some women were surprised by their UI the majority of pregnant women accepted their UI as part of being pregnant and think it will resolve after delivery. This belief is based on information women gathered in a variety of ways and is in line with other studies (Moossdorff-Steinhauser et al., 2021b, c). The women did not experience their UI as very bothersome and indicated that they would seek help if there was an increase in the amount and frequency of urine loss, occurrence at unexpected moments or an increase in negative feelings regarding their UI. This is in line with the results of two Dutch studies reporting on help-seeking behavior of pregnant and post-partum women (Moossdorff-Steinhauser et al., 2021b, c). No participant in this study had visited a specialized PT to start pelvic floor muscle exercises. Although some women asked their HCP about their UI, the majority did not. Nonetheless, several women said that they would talk about it if they were asked. Only for some an open question like ‘do you have any other issues you would like to discuss’ was a trigger to discuss UI. In a study by Buurman et al. women stated that if a HCP did not ask about UI, it could not be that bad (Buurman & Lagro-Janssen, 2013). The HCP’s in the focus group reported that a question on UI in pregnancy and post-partum is not standard and this could potentially mean that women are not well informed about UI In general, the women we interviewed seem not to be bothered to a large extent by UI. The women notice, are surprised by, and dislike UI symptoms, yet they are inclined to accept UI as “normal” and being part of pregnancy and delivery. It does not have a large impact on their daily lives and they deploy several strategies to deal with it. To our knowledge is not known if the level of bother of peri-partum women regarding UI rises if they are informed routinely regarding UI, i.e. prevalence, recovery and treatment options.

As the women also noted that they read contradicting information and advice on the internet, the HCP could be the one to provide reliable information. The HCP’s should provide women with realistic expectations regarding post-partum UI and discuss treatment options. However, the HCP’s experienced the duration of the consultation as an important barrier to discuss UI as well as the lack of a standard question in the EPR. One of the reasons a standard question on UI is not in the EPR might be that in guidelines regarding pre- and postnatal care in The Netherlands, UI is hardly mentioned (Koninklijke Nederlandse Organisatie van Verloskundigen (KNOV), 2018; Nederlands Huisartsen Genootschap (NHG), 2018; Nederlandse Vereniging voor Obstetrie en Gynaecologie (NVOG), 2014). The NICE guideline on antenatal care for uncomplicated pregnancies recommends specifically to discuss pelvic floor muscle exercises, ideally at 10 weeks gestation (National Institute for Health and Care Excellence (NICE), 2019a). Likewise, it is recommended to review and adapt the Dutch peri-partum guidelines, in co-creation with the involved professionals, with regard to creating awareness of UI and optional interventions such as specialized physical therapy.

According to the HCP’s, women could be informed through a leaflet or on televisions/screens in the waiting room. Other suggestions put forward were discussing UI with a specialized nurse who is already seeing the women to discuss the delivery or with a specialized PT. This is an interesting option because in that case there would also be an opportunity to provide the women with reliable information and pelvic floor muscle exercises. This will stimulate self-efficacy. Specialized PT is a first line treatment option for UI, recommended by the NICE guidelines (National Institute for Health and Care Excellence (NICE), 2019a, b). However, especially the gynecologists and resident’s showed some reservations regarding the effect of specialized PT. This is in line with the results of a Cochrane review which stated that the effect of pelvic floor muscle exercises ante- or post-natal for the treatment of UI is still uncertain (Woodley et al., 2020). But we need to keep in mind that these results are based on (small) studies of (very) low quality. Interestingly, another Cochrane review on the effect of pelvic floor muscle exercises for women with UI in the general population concluded that pelvic floor muscle exercises can cure and improve symptoms of UI (Dumoulin et al., 2018). Therefore, it might be justified to consider offering peri-partum women pelvic floor muscle exercises and in the meantime executing high quality randomized controlled trials to support evidence for this strategy. A midwife who works closely with a specialized PT in her practice mentioned the benefits of this collaboration. Also other issues like finding a properly qualified specialized PT were mentioned. This reveals that there might be a knowledge gap that needs to be addressed.

## Limitations

Our study has several limitations. The focus group consisted of proportionally more HCP’s who work in one hospital and not in primary perinatal care. In The Netherlands only women with high risk complicated pregnancies are monitored by a HCP in a hospital and low risk pregnancies by a midwife in primary care. As a result these consultations might have a different focus. Inclusion of women was based on self-selection and therefore women for whom discussing UI is very difficult or a taboo might not have expressed interest. All women but one had a Dutch cultural background and came from the south of The Netherlands. Fourteen percent of women living in The Netherlands have a non-western migration background (Centraal Bureau voor Statistiek (CBS), 2021). This group might have different beliefs and experiences regarding peri-partum UI. Therefore the results presented in this study are not transferable to these women. Moreover, the interviewed pregnant women were mostly higher educated (Centraal Bureau voor Statistiek (CBS), 2020). Generalizability of the results is limited due to the qualitative design of this study. However, due to the variety of the sample and the richness of the interviews, valuable knowledge regarding the beliefs and experiences of pregnant and up to 1 year post-partum women with UI and HCP’s has emerged. We therefore think that the insights gained might transferable to women in similar contexts.

## Conclusions

The 14 interviewed pregnant and up to 1 year post-partum women with UI are hardly bothered by their UI and accept it as part of being pregnant or as a result of the delivery. This belief probably originates from information from a HCP, friend or the internet. Some women ask their HCP about their UI but when not asked some women do not disclose their UI. HCP’s do not discuss UI on a standard basis. Discussing UI can empower women and contribute to their self-efficacy.

## Data Availability

Upon request (in Dutch).

## Code Availability

Upon request (in Dutch).

## References

[CR1] Borello-France D, Burgio KL, Richter HE, Zyczynski H, Fitzgerald MP, Whitehead W, Fine P, Nygaard I, Handa VL, Visco AG, Weber AM, Brown MB (2006). Fecal and urinary incontinence in primiparous women. Obstetrics and Gynecology.

[CR2] Braun V, Clarke V (2006). Using thematic analysis in psychology. Qualitative Research in Psychology.

[CR3] Burgio KL, Zyczynski H, Locher JL, Richter HE, Redden DT, Wright KC (2003). Urinary incontinence in the 12-month postpartum period. Obstetrics and Gynecology.

[CR4] Buurman MB, Lagro-Janssen AL (2013). Women’s perception of postpartum pelvic floor dysfunction and their help-seeking behaviour: A qualitative interview study. Scandinavian Journal of Caring Sciences.

[CR5] Centraal Bureau voor Statistiek (CBS). (2020). *Leerlingen, deelnemers en studenten*. Retrieved May 05 from https://opendata.cbs.nl/statline/#/CBS/nl/dataset/71450ned/table?fromstatweb

[CR6] Centraal Bureau voor Statistiek (CBS). (2021). *Hoeveel mensen met een migratieachtergrond wonen in Nederland*. Retrieved May 05 from https://www.cbs.nl/nl-nl/dossier-asiel-migratie-en-integratie/hoeveel-mensen-met-een-migratieachtergrond-wonen-in-nederland

[CR7] Cooke CM, O'Sullivan OE, O'Reilly BA (2018). Urogynaecology providers’ attitudes towards postnatal pelvic floor dysfunction. International Urogynecology Journal.

[CR8] Diez-Itza I, Zubikarai M, Galan C, Ginto L, Saro J, Arrue M (2020). Factors involved in the persistence of stress urinary incontinence from postpartum to 12 years after first delivery. Neurourology and Urodynamics.

[CR9] Dumoulin C, Cacciari LP, Hay-Smith EJC (2018). Pelvic floor muscle training versus no treatment, or inactive control treatments, for urinary incontinence in women. Cochrane Database of Systematic Reviews.

[CR10] Hägglund D, Walker-Engström ML, Larsson G, Leppert J (2001). Quality of life and seeking help in women with urinary incontinence. Acta Obstetricia Et Gynecologica Scandinavica.

[CR11] Hansen BB, Svare J, Viktrup L, Jørgensen T, Lose G (2012). Urinary incontinence during pregnancy and 1 year after delivery in primiparous women compared with a control group of nulliparous women. Neurourology and Urodynamics.

[CR12] Koch LH (2006). Help-seeking behaviors of women with urinary incontinence: An integrative literature review. Journal of Midwifery & Women's Health.

[CR13] Koninklijke Nederlandse Organisatie van Verloskundigen (KNOV). (2018). *Multidisciplinaire richtlijn postnatale zorg*. Retrieved Mai 05 from https://www.knov.nl/kennis-en-scholing/richtlijnen-en-standaarden/richtlijn?componentid=6881288&title=Postnatale%252bzorg%252b(multidisciplinaire%252brichtlijn)

[CR14] Martínez Franco E, Parés D, LorenteColomé N, Méndez Paredes JR, AmatTardiu L (2014). Urinary incontinence during pregnancy. Is there a difference between first and third trimester?. European Journal of Obstetrics, Gynecology, and Reproductive Biology.

[CR15] Martin-Martin S, Pascual-Fernandez A, Alvarez-Colomo C, Calvo-Gonzalez R, Muñoz-Moreno M, Cortiñas-Gonzalez JR (2014). Urinary incontinence during pregnancy and postpartum. Associated risk factors and influence of pelvic floor exercises. Archivos Espanoles de Urologia.

[CR16] Moossdorff-Steinhauser HFA, Berghmans BCM, Spaanderman MEA, Bols EMJ (2021). Prevalence, incidence and bothersomeness of urinary incontinence in pregnancy: A systematic review and meta-analysis. International Urogynecology Journal.

[CR17] Moossdorff-Steinhauser HFA, Berghmans BCM, Spaanderman MEA, Bols EMJ (2021). Urinary incontinence 6 weeks to 1 year post-partum: Prevalence, experience of bother, beliefs, and help-seeking behavior. International Urogynecology Journal.

[CR18] Moossdorff-Steinhauser HFA, Berghmans BCM, Spaanderman MEA, Bols EMJ (2021). Urinary incontinence during pregnancy: Prevalence, experience of bother, beliefs, and help-seeking behavior. International Urogynecology Journal.

[CR19] National Institute for Health and Care Excellence (NICE). (2019b). *Urinary incontinence and pelvic organ prolapse in women: management (NG123)*. Retrieved May 05 from https://www.nice.org.uk/guidance/ng123/chapter/chapter/Recommendations#physical-therapies 31211537

[CR20] National Institute for Health and Care Excellence (NICE). (2019a). *Antenatal care for uncomplicated pregnancies (CG62)*. Retrieved May 05 from https://www.nice.org.uk/guidance/cg62/resources/antenatal-care-for-uncomplicated-pregancies-pdf-975564597445 31961630

[CR21] Nederlands Huisartsen Genootschap (NHG). (2018). *Zwangerschap en Kraamperiode*. Retrieved May 05 from https://richtlijnen.nhg.org/standaarden/zwangerschap-en-kraamperiode

[CR22] Nederlandse Vereniging voor Obstetrie en Gynaecologie (NVOG). (2014). *Samenvattingskaart Richtlijn Urine-incontinentie in de tweede- en derde lijnszorg 2.0*. Retrieved May 05 from https://www.nvog.nl/wp-content/uploads/2018/02/Samenvattingskaart-richtlijn-update-urine-incontinentie-definitief-versie-juni-2014-e.pdf

[CR23] Rortveit G, Hannestad YS, Daltveit AK, Hunskaar S (2001). Age- and type-dependent effects of parity on urinary incontinence: The Norwegian EPINCONT study. Obstetrics and Gynecology.

[CR24] Tong A, Sainsbury P, Craig J (2007). Consolidated criteria for reporting qualitative research (COREQ): A 32-item checklist for interviews and focus groups. International Journal for Quality in Health Care.

[CR25] Wagg AR, Kendall S, Bunn F (2017). Women’s experiences, beliefs and knowledge of urinary symptoms in the postpartum period and the perceptions of health professionals: A grounded theory study. Primary Health Care Research & Development.

[CR26] Woodley SJ, Lawrenson P, Boyle R, Cody JD, Mørkved S, Kernohan A, Hay-Smith EJC (2020). Pelvic floor muscle training for preventing and treating urinary and faecal incontinence in antenatal and postnatal women. Cochrane Database Syst Rev.

